# Theoretical investigations of oxygen vacancy effects in nickel-doped zirconia from ab initio XANES spectroscopy at the oxygen K-edge

**DOI:** 10.3762/bjnano.13.85

**Published:** 2022-09-15

**Authors:** Dick Hartmann Douma, Lodvert Tchibota Poaty, Alessio Lamperti, Stéphane Kenmoe, Abdulrafiu Tunde Raji, Alberto Debernardi, Bernard M’Passi-Mabiala

**Affiliations:** 1 Groupe de Simulations Numériques en Magnétisme et Catalyse, Faculté des Sciences et Techniques, Université Marien Ngouabi, B.P. 69 Brazzaville, Congo; 2 IMM-CNR, Unit of Agrate Brianza, via C. Olivetti 2, 20864 Agrate Brianza (MB), Italy; 3 Department of Theoretical Chemistry, University of Duisburg-Essen, Universitätstr. 2, D-45141 Essen, Germany; 4 Department of Physics, College of Science, Engineering and Technology (CSET), University of South Africa (UNISA-Florida Campus), Corner of Christiaan de Wet Road & Pioneer Avenue, Florida 1709, South Africa; 5 National Institute of Theoretical and Computational Sciences (NITheCS), University of South Africa (UNISA), Preller St, Muckleneuk, Pretoria 0002, South Africa; 6 Unité de Recherche en Nanomatériaux et Nanotechnologies, Institut National de Recherche en Sciences Exactes et Naturelles (IRSEN), Brazzaville, Congo

**Keywords:** defect, ligand field, nickel, oxidation state, oxides, spectroscopy, spintronics, vacancy, X-ray absorption, X-ray absorption near-edge structure (XANES), zirconia

## Abstract

In this study, we present theoretical X-ray absorption near-edge structure (XANES) spectra at the K-edge of oxygen in zirconia containing Ni dopant atoms and O vacancies at varying concentrations. Specifically, our model system consist of a supercell composed of a zirconia (ZrO_2_) matrix containing two nickel dopants (2Ni), which substitute two Zr atoms at a finite separation. We found the 2Ni atoms to be most stable in a ferromagnetic configuration in the absence of oxygen vacancies. In this system, each Ni atom is surrounded by two shells of O with tetrahedral geometry, in a similar way as in bulk cubic zirconia. The oxygen K-edge XANES spectrum of this configuration shows a pre-edge peak, which is attributable to dipole transitions from O 1s to O 2p states that are hybridized with unoccupied Ni 3d states. The intensity of this pre-edge peak, however, reduces upon the introduction of a single vacancy in the 2Ni-doped zirconia matrix. The corresponding ground state remains ferromagnetic, while one of the nickel atoms adopts a trigonal bipyramidal geometry, and the other one remains in a tetrahedral geometry. Furthermore, the introduction of two vacancies in the 2Ni-doped zirconia results in the two Ni atoms having distorted octahedral and trigonal bipyramidal geometries and being coupled antiferromagnetically in the ground state. Additionally, the oxygen K-edge XANES spectrum shows a further decrease in the intensity of the pre-edge peak, compared to the case of a single vacancy. Thus, the changes in the intensity of the pre-edge peak evidence a major structural change in the local environment around nickel atoms and, by extension, in the zirconia matrix. This change is due to the structural disorder induced by the 2Ni dopants and the O vacancies. Furthermore, the analysis of the XANES signatures shows that the oxidation state of nickel atoms changes with the introduction of oxygen vacancies. Our study therefore shows a possibility to control the oxidation state and magnetic order in a typical diluted magnetic oxide. Such a finding may be crucial for spintronics-related applications.

## Introduction

The search for room-temperature magnetic semiconductors has been the driving force behind the increasing interest of material scientists and solid-state physicists in magnetic oxides [1]. This is due to their potential applications as building block of spintronic devices. Magnetic oxides are semiconducting or insulating materials doped randomly or uniformly with magnetic impurities in the oxide matrix. A typical example are thin films of uniformly Fe-doped ZrO_2_ where dopant concentrations, *x*, from the diluted regime (i.e., *x* = 1–5 atom % [2]) to very high concentrations (up to *x* ≈ 25 atom %) have been achieved [3–4]). Also, in the last decade, considerable efforts have been devoted to determine the type of magnetic interaction (paramagnetic, ferromagnetic, or antiferromagnetic) and the microscopic mechanisms responsible for the magnetism in doped magnetic oxides (DMO) [5]. Several experimental [6–10] and theoretical [7,11–13] studies that investigated DMO showed the crucial role of intrinsic defects in determining the magnetic properties [14–19]. Among the DMO that have been investigated, transition metal-doped zirconia [20–23], a super-hard oxide which is being currently employed in ultra-scaled electronics for its high dielectric constant [24–25] have received significant attention because of its practical applications. Thus, recently, exploiting first principles simulations and X-ray absorption near edge spectroscopy (XANES) in high magnetic fields, Ciprian et al. [4] showed that in Fe-doped ZrO_2_, the magnetism is produced by an oxygen-mediated super-exchange mechanism between Fe dopants, controlled by O vacancies. This finding also suggest the possibility that the microscopic mechanism underpinning this conclusion [4] may be common to a large set of DMO. It is thus important to investigate other types of dopants in DMO or other types of oxide matrices doped with transition metals.

Oxygen vacancies play a fundamental role in the aforementioned microscopic mechanisms since an excess or lack of oxygen vacancies with respect to their stoichiometric concentration required to ensure overall charge neutrality can modify the presence of defect states in the electron bandgap [4,26–27]. In diluted magnetic semiconductors (DMS), magnetic impurities such as the transition metals (TM) Fe or Ni are introduced to produce a magnetic ground state. The TM atom usually occupies the Zr site substitutionally. However, due to the difference in the oxidation state of the TM atom and Zr, oxygen vacancies are created to maintain an overall charge neutrality. This is aptly demonstrated in the experimental investigations of Fe-doped zirconia using X-ray photoelectron spectroscopy (XPS) [26], XANES spectra [27], and synchrotron radiation measurements [4], where it was suggested that for every two dopant atoms of Fe in zirconia, one single O vacancy is created. It is, thus, expected that in similarity to the effect of Fe doping in zirconia, substitutional Ni in zirconia will also create oxygen vacancies, although of different concentration due to the different oxidation states of Fe and Ni. Furthermore, O vacancies may be inadvertently introduced into semiconductors as a result of the processing conditions. For example, in a semiconductor manufacturing process, ambient thermodynamic conditions such as oxygen pressure and post-annealing atmosphere are directly connected to the formation and concentration of oxygen vacancies [28].

The presence of the O vacancy modifies the oxidation state of the substitutional transition metal atom and thus the magnetic properties of the zirconia system due to the variation of the number of electrons in the transition metal d orbital states. In this context, an experimental probe sensitive to the oxidation states of the magnetic ion as well as the local environment specific to a probed atom will be vital. Thus, X-ray absorption near-edge structure (XANES) spectroscopy becomes relevant since its signature can indirectly reveal the presence of O vacancies in a metal oxide matrix. It is therefore a technique useful to understand the microscopic origin of magnetism in DMO. XANES spectroscopy concerns the part of the X-ray absorption spectrum near the ionization threshold of core electrons with orbital momentum *l* belonging to the absorbing atom [29]. It is sensitive to the electronic structure of the absorbing atom, since its intensity is nearly proportional to the density of the unoccupied states whose symmetry verifies the electric dipolar selection rule Δ*l* = ±1, and also to the stereochemical arrangement of neighbors around the absorbing atom. By tuning the incident photon energy to the X-ray edge energy of the target atom, it is possible to determine the coordination environment, bonding characteristics, as well as spin and oxidation states of the atom in the sample [30]. In the case of transition metal atoms, L*_2,3_*-edge XAS is particularly suitable for probing 3d valence orbitals via the dipole-allowed 2p–3d transitions. Several factors may affect the L*_2,3_*-edge spectrum, including the structure of the metal complex, the covalency of the metal–ligand bonds, and the change in the metal oxidation state. The latter may lead to distinct changes in the spectral shape and incident energy [30–31]. For high-spin transition metals, for instance, it has been established that the L*_2,3_*-edge spectrum shifts to higher energies with increasing formal metal oxidation state and shows significant changes in spectral shape [30–32].

Accessing the oxidation state information via the calculation of L*_2,3_*-edge spectra for localized final states in systems such as transition metal elements, that is, with strong electron–electron interactions, using single-electron approaches based on density functional theory (DFT) may not be an easy task. In fact, the 2p hole and 3d hole radial wave functions in such systems overlap significantly and may render the reproduction of the experimental spectrum difficult [33]. However, it is possible to get such information via the ligand K-edge, with the possibility of scanning unoccupied 3d metal states since they are hybridized with the 2p ligand states, as we demonstrated in our earlier work on the O K-edge in iron-doped zirconia (ZrO_2_:Fe) [27]. There, we compared the experimental XANES data with the corresponding first principle spectra obtained using the ab initio technique developed by Gougoussis and co-workers [34]. In this previous work [27], we considered Fe dopant atoms in the +3 oxidation state and at varying concentrations *x* (*x* = 6–25 atom %) in ZrO_2_[27]. We found that substituting Zr with Fe atoms leads to a radical change in the O K-edge XANES spectrum, especially in the pre-edge region where a pre-edge peak appears. This pre-edge peak is ascribed to dipole transitions from O 1s to O 2p states that are hybridized with unoccupied Fe 3d states. Furthermore, we also observed an increase of this pre-edge peak with Fe concentration, which may indicate an increase of unoccupied 3d states in the system.

In the present work, we expand the study of transition metal doping of ZrO_2_ by considering another magnetic dopant, namely nickel (Ni), which has oxidation states similar to those of Fe but with two more electrons in the d shell than Fe. It is thus expected that a Ni-doped ZrO_2_ matrix (i.e., Ni*_x_*Zr*_1−x_*O_2_) may have electronic properties different from Fe-doped zirconia. We note that zirconia has two stable phases, that is, monoclinic baddeleyite and a cubic distorted fluorite structure. The fluorite structure is not stable under ambient conditions and, thus, zirconia is usually found in the monoclinic phase. The cubic distorted fluorite structure may however be stabilized by a doping or by deposition as a thin film (because the surface energy of the cubic structure is lower than that of the monoclinic structure) [35]. Thus, our structural phase of reference in this work is cubic distorted fluorite zirconia since we are doping it and also due to the fact that actual experiments involving atomic layer deposition of TM-doped zirconia thin films revealed the cubic phase [26]. In the distorted fluorite structure, each Zr atom is arranged in a fcc lattice and is surrounded by a nearest-neighbor shell of four oxygen atoms at the vertices of a tetrahedron having Zr in the center. The next-nearest-neighbor shell consists of four additional oxygen atoms at the vertices of a second tetrahedron also having Zr in its center. To model Ni-doped zirconia, we substituted two Ni dopant atoms at two Zr sites and then varied the number of O vacancies. It should be noted that this number of Ni dopant atoms implies a concentration of 6.25 atom % Ni in our model system. The introduction of O vacancies introduces atomic disorder into the system, which may affect the oxidation state of the Ni dopant atoms. Also, XANES spectra features have been shown to be sensitive to the onset or presence of structural disorder in a crystalline structure [36–37]. Thus, we have performed calculations of the XANES K-edge of oxygen atoms in Ni-doped zirconia to determine the signatures of disorder introduced by the O vacancies. Also, by combining the XANES signatures with the projected density of states (PDOS), we are able to determine the oxidation state of Ni dopant atoms in zirconia. The XANES calculations have been performed using the single-electron DFT framework as proposed by Gougoussis and co-workers [34].

The paper is organized as follows: In the following section, we present the computational parameters and structural models used in the calculations. Then, the results of computed spectra of the O K-edge in nickel-doped zirconia with and without O vacancies are presented and discussed. A conclusion is given in the final section.

## Computational Methodology

### Structural relaxation

First principles calculations have been performed with the Quantum-ESPRESSO code [38] using the plane waves basis set in the pseudopotential approach and periodic boundary conditions. We used the ultrasoft pseudopotentials and the generalized gradient approximation (GGA) in the parametrization of Perdew, Burke, and Ernzerhof (PBE) for the exchange–correlation functional [39]. The Brillouin zone is sampled using a 2 × 2 × 2 Monkhorst–Pack grid, while convergence thresholds of 10^−8^ Ry and 10^−3^ Ry/Bohr have been adopted for the total energy and atomic forces, respectively. The plane wave basis set used to represent the Kohn–Sham (KS) orbitals has a kinetic energy of 50 Ry with 500 Ry as the charge density. The initial system for the calculations consists of a tetragonal supercell of ZrO_2_ containing 96 atoms (64 O atoms and 32 Zr atoms), which eventually relaxes to an approximately cubic supercell as an effect of doping (see [4,40–41] for Fe-doped ZrO_2_ and [42] for Ge-doped HfO_2_, a similar oxide, where a systematic study of the transition from the tetragonal to the cubic supercell is presented).

**Figure 1:**
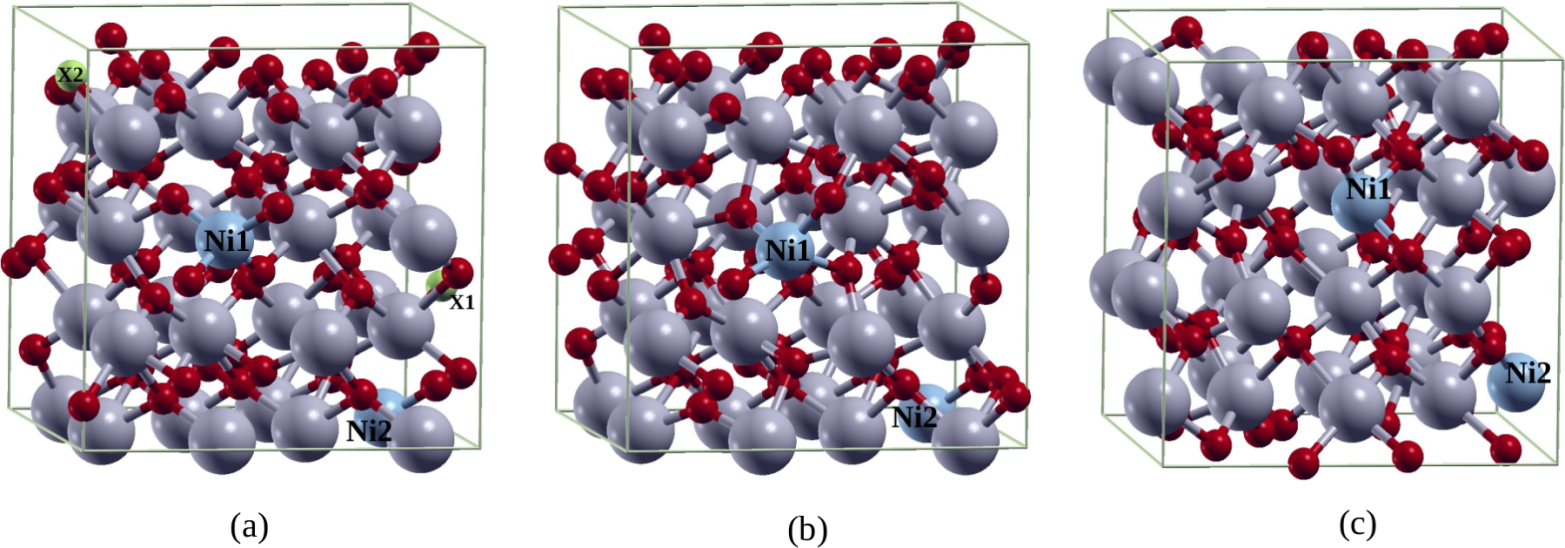
(a) Zirconia containing two Ni atoms substituting Zr atoms without O vacancies, that is, structure S_0_. (b) Structure S_1_, which is S_0_ containing a single O vacancy wherein the oxygen atom (green sphere) marked X1 in (a) has been removed. (c) Structure S_2_, which is S_0_ containing a double O vacancy wherein the O atoms (green spheres) marked X1 and X2 have been removed.

To model the nickel-doped system, we substitute two Zr atoms at their respective lattice sites with two nickel atoms, Ni1 and Ni2. This corresponds to 6.25 atom % dopant concentration of the 2Ni-doped zirconia. Spin-polarized structural relaxation calculations are then performed to determine the energetically most stable spin configuration of the Ni dopant atoms in zirconia. The relaxed configuration of ZrO_2_ with the Ni1 and Ni2 dopants containing no vacancies, that is, the S_0_ structure is cubic. This structure is shown in Figure 1a. The observation of cubic cell symmetry as the ground state in 2Ni-doped zirconia is consistent with the observation in 2Fe-doped zirconia [4]. Single and double oxygen vacancies are then introduced into the S_0_ structure in such a way that the vacancies are sited at the farthest possible separation from the dopants. This is to minimize as much as possible the distortion of the Ni symmetry in zirconia due to the presence of vacancies. The two oxygen atoms to be removed successively for the construction of Ni-doped ZrO_2_ containing single and double vacancies are represented by green spheres marked as X1 and X2 in Figure 1a. These new structures containing single and double vacancies are denoted as S_1_ and S_2_ respectively. Similar to S_0_, their relaxed configurations remain cubic as shown in Figure 1. Since oxygen vacancies introduce disorder in the system, the two structures S_1_ and S_2_ enable us to investigate the effect of disorder on the structures and electronic properties of Ni-doped zirconia. X-ray absorption near-edge structure (XANES) offers a deep insight into the electronic structure of materials as we shall show in the following sections.

### XANES calculation

The XANES spectra are computed using the XSPECTRA code [34], which is a module in the Quantum-ESPRESSO computational package. In the code, the X-ray absorption cross section is modeled in terms of a transition operator coupling initial and final states, which are solutions of the KS equations. For the K-edge calculation, the initial state is a core 1s orbital calculated from an isolated absorbing atom in the absence of a core hole, while the final state is obtained self-consistently through the solution of the KS equations for the whole system while including the core hole effects in the pseudopotential of the absorbing atom [34]. Within this pseudopotential approach, the final all-electron wave function is reconstructed from the pseudowave function by means of the projector augmented wave method [43]. The isotropic cross section has been found necessary to calculate XANES spectra, since our relaxed structural phase of Ni-doped zirconia is cubic. For a general symmetry, the isotropic electric dipole cross section is obtained by a linear combination of three cross sections calculated along three perpendicular directions of polarization, namely σ(0,0) = 1/3(σ*_xx_* + σ*_yy_* + σ*_zz_*) [44]. In practice, the cross section is calculated as follows: First, the charge density is obtained through a self-consistent DFT calculation with 1s core hole on the absorbing oxygen atom, and the cross section is then calculated for a given polarization direction using the Lanczos method and the continued fraction [45]. This approach does not require an explicit calculation of empty states and is very fast since only the charge density is needed [34]. Our supercell of size 2 × 2 × 2 (96 atoms) is sufficiently large to avoid the interaction between periodic images of the absorbing atom. In fact, a smaller supercell size of 72 atoms has been used for the similar SiO_2_ and has been found to produce theoretical spectra that are consistent with experimental data [45]. Also, a Lorentzian convolution with a variable broadening parameter γ has been applied in the continued fraction. For this purpose, we used γ = 0.3 eV for photon energies up to 1.5 eV and γ = 0.8 eV for photon energies above 10 eV, with a linear variation in the intermediate photon range. These parameters are equal to those used in our previous ab initio calculations to reproduce experimental O K-edge XANES spectra in the case of iron-doped zirconia [27]. We regard the extrapolation of these previous parameters to the present work as reasonable, since both Ni and Fe are magnetic transition metal elements with similar oxidation states, although Ni has two more electrons in its d shell than Fe.

## Results and Discussion

**Figure 2:**
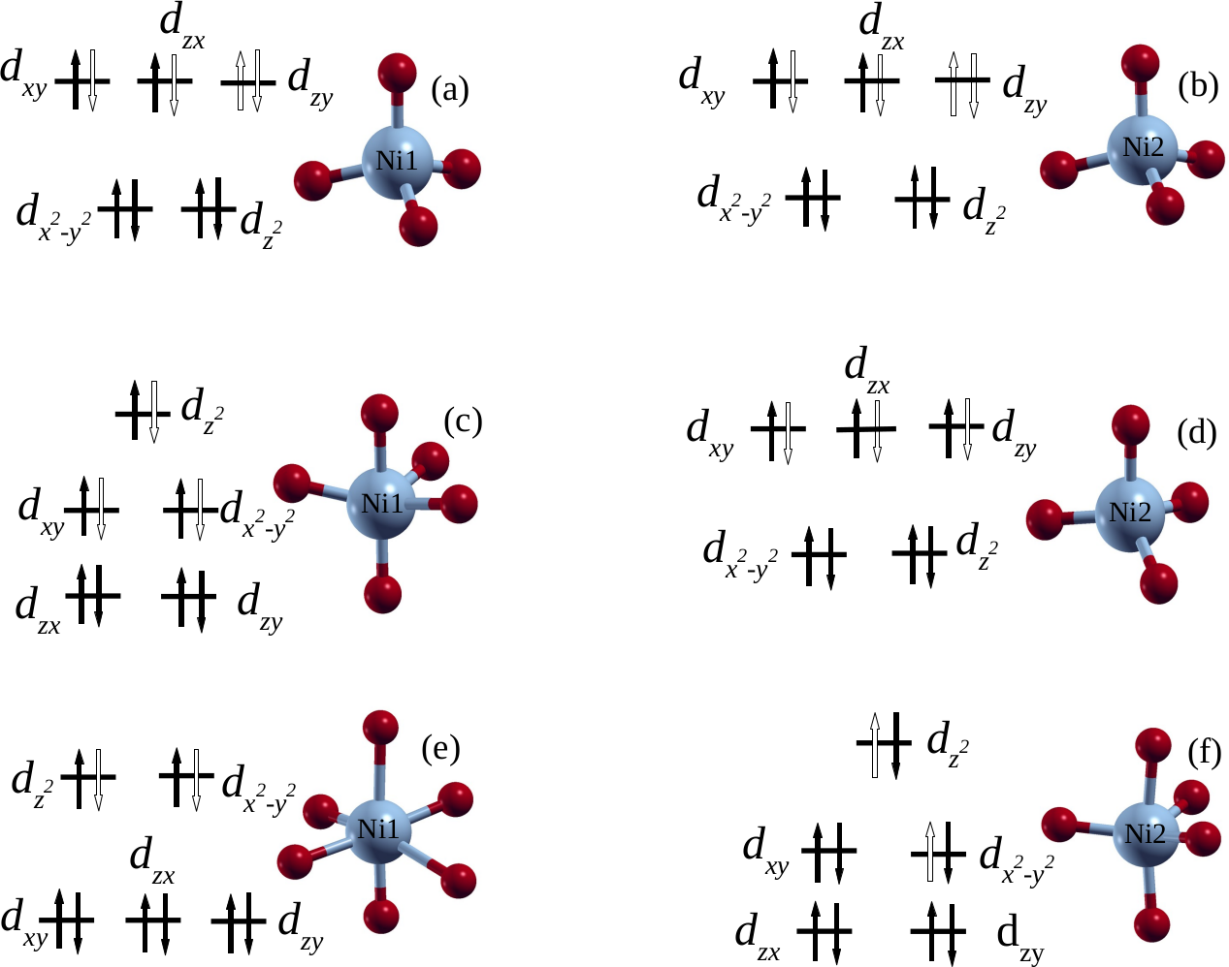
Relaxed nickel geometries and crystal-field splitting diagrams for different Ni-doped zirconia containing varying oxygen vacancy concentrations. (a, b) Nickel dopant atoms in tetrahedral geometry in the structure S_0_ with Ni1 and Ni2 being spin-up polarized. It should be noted that S_0_ has no oxygen vacancies. (c, d) Nickel dopant atoms in trigonal bipyramidal and tetrahedral geometries in the structure S_1_ with Ni1 and Ni2 being spin-up polarized. Structure S_1_ has one oxygen vacancy. (e, f) Nickel dopant atoms in octahedral and trigonal bipyramidal geometries in the structure S_2_ with Ni1 being spin-up and Ni2 being spin-down polarized. Structure S_2_ has two oxygen vacancies. In the figures, blue and red spheres represent Ni and O atoms, respectively. Black filled arrows represent occupied electron states while the hollow arrows indicate empty electron states.

Before discussing the XANES spectra for the doped and non-stoichiometric ZrO_2_, its is important to describe the relaxed structures of S_0_, S_1_, and S_2_ containing Ni^4+^ ions (Figure 2a,b), Ni^3+^ ions (Figure 2c,d), and Ni^2+^ ions (Figure 2e,f), respectively, since these are the structures used for the XANES calculations. We have found that in the relaxed structure of S_0_, ferromagnetic spin alignments are more stable compared to the antiferromagnetic configuration, and the local environment around Ni dopants assumes to a tetrahedral symmetry following the relaxation (Figure 2a,b). In the case of S_1_, the ferromagnetic configuration is still the most stable while the site symmetries of Ni1 and Ni2 show a coexistence of distorted tetrahedral and trigonal bipyramidal geometries, respectively (Figure 2c,d). However, in the case of S_2_, the antiferromagnetic spin configuration is the most stable in which the nickel atoms Ni1 and Ni2 coexist in distorted octahedral and trigonal bipyramidal geometries, respectively (Figure 2e,f).

**Figure 3:**
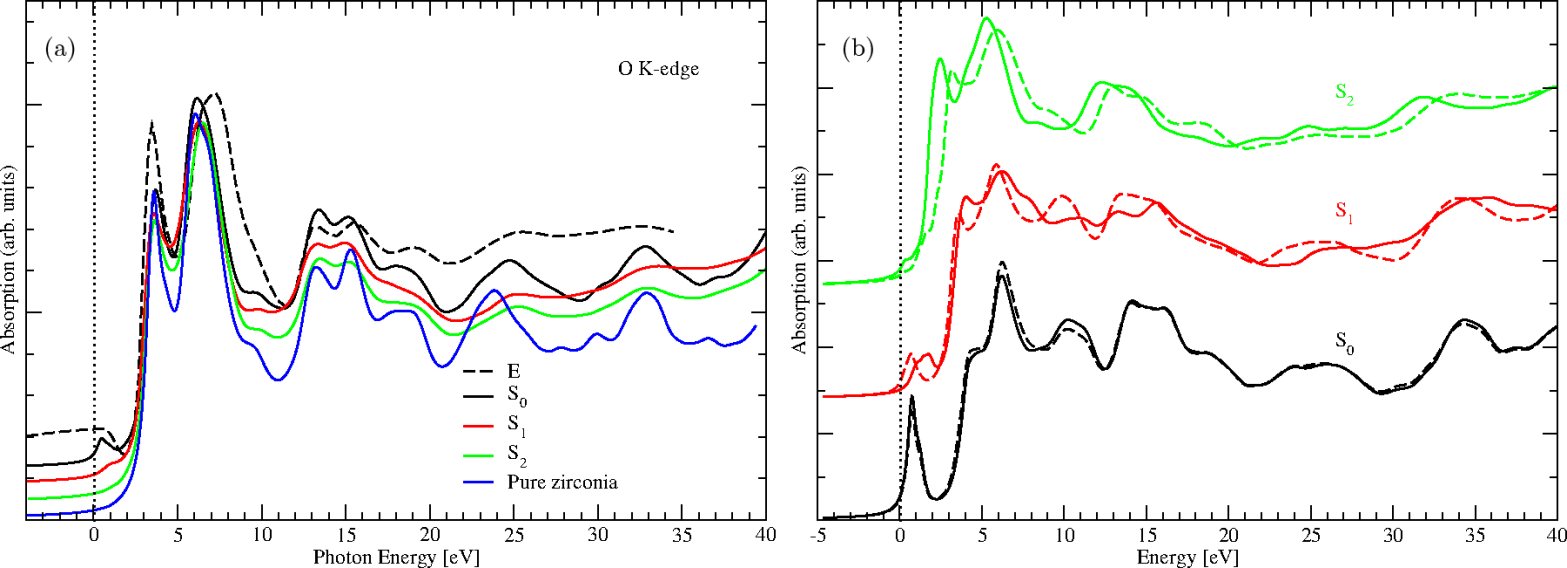
(a) O K-edge spectra of pure zirconia (blue curve) and of nickel-doped zirconia (ZrO_2_:Ni) at *x* = 6.25 atom % Ni concentration and varying number of vacancies. The notations S_0_, S_1_, and S_2_ correspond to the doped structures containing zero, one, and two oxygen vacancies, respectively. Note the presence of the pre-edge peak, which decreases when the number of oxygen vacancies increases. Also, the black, red, and green curves are the XANES spectra obtained from the averages of 64, 63, and 62 individual O K-edge spectra in the zirconia supercell, while the spectrum E (black dashed line) corresponds to the experimental O K-edge spectrum in iron-doped zirconia (ZrO_2_:Fe) at *x* = 6 atom % Fe concentration from [27]. (b) The mean contributions of the first oxygen shells around the nickel atoms Ni1 (solid lines) and Ni2 (dashed lines) to the O K-edge spectra of the structures S_0_, S_1_, and S_2_.

We present XANES spectra for defect-free zirconia, that is, without Ni dopant (*x* = 0 atom %), and the doped structures S_0_, S_1_, and S_2_ in Figure 3a. In the case of defect-free zirconia, the XANES spectrum is needed only for one oxygen atom site since the lattice sites of all the oxygen atoms are equivalent. The resulting spectrum shows no peak in the pre-edge region where the Fermi level is considered as zero energy in the plot (Figure 3a). This observation is in agreement with our previous observations in the case of iron-doped zirconia [27]. As noted earlier, when the two nickel atoms are introduced (structure S_0_), the ferromagnetic structure is the more stable configuration, and the O K-edge spectrum, calculated as the average of contributions from all oxygen atoms in the supercell (i.e., spectra from the 64 O atoms), shows a pre-edge peak in the energy range between 0 and 1.45 eV (Figure 3a). This pre-edge peak is ascribed to dipole transitions from 1s to 2p states that are hybridized with unoccupied nickel 3d states as we demonstrated previously in the case of iron-doped zirconia [27]. For comparison with experimental data, we have also plotted the experimental O K-edge spectrum of iron-doped zirconia (ZrO_2_:Fe) at *x* = 6 atom % Fe dopant concentration from [27] (Figure 3a). This previous experimental spectrum is very similar to the current theoretical ones since the Fe has oxidation states similar to Ni, although Fe has two electrons less in the d shell. It has to be noticed that, in the previous study, we have considered iron atoms in the oxidation state +3.

**Figure 4:**
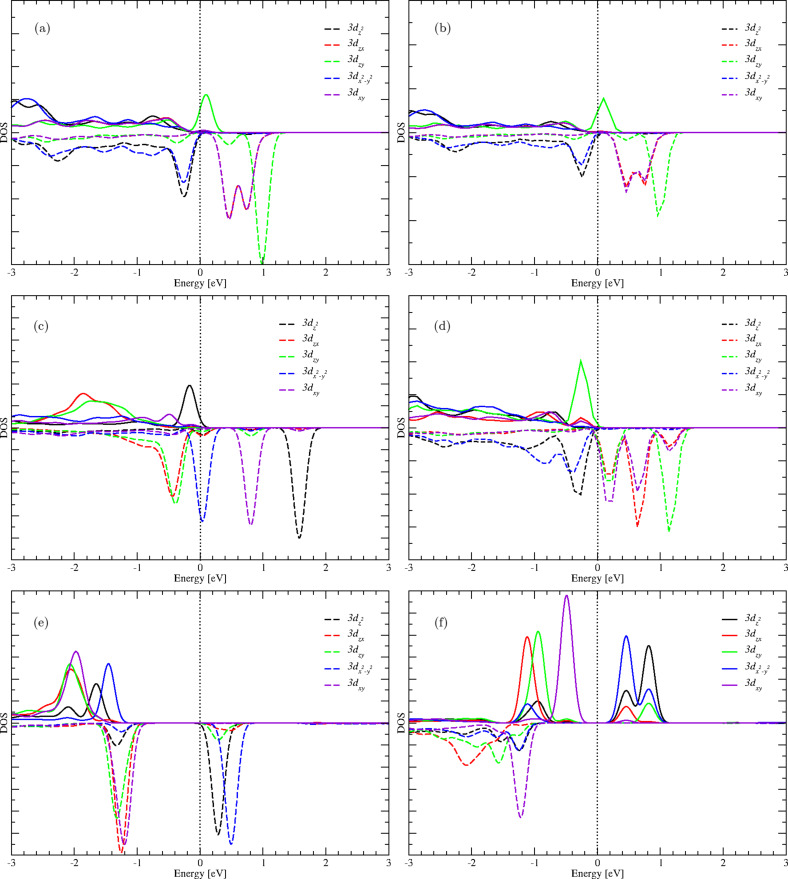
Projected densities of states (PDOS) of nickel atoms Ni1 and Ni2. The solid line corresponds to spin-up polarization, the dashed line corresponds to spin-down. (a, b) Nickel atoms Ni1 (spin-up) and Ni2 (spin-up) in tetrahedral geometries, belonging to the doped structure S_0_. (c, d) Nickel atoms Ni1 (spin-up) and Ni2 (spin-up) in trigonal bipyramidal and tetrahedral geometries, respectively, belonging to the doped structure S_1_. (e, f) Nickel atoms Ni1 (spin-up) and Ni2 (spin-down) in octahedral and trigonal bipyramidal geometries, respectively, belonging to the doped structure S_2_.

Assuming the nickel atoms to be in the oxidation state +4 since they substitute Zr^4+^ cations and considering the nickel atom Ni1, we observe that its contribution to the pre-edge peak is mainly due to its 3d*_xy_*, 3d*_zx_*, and 3d*_zy_* spin-down orbitals, as well as its 3d*_zy_* spin-up orbitals, as shown by the PDOS in Figure 4a. Ni1 with Ni^4+^ oxidation state and 3d^6^ orbital configuration has a tetrahedral coordination, which suggests that it is in low spin-polarization with an electron configuration 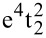 according to ligand field theory [46–48] (cf. Figure 2). This is because the two orbitals e and t_2_ participate together to the transitions occurring in the pre-edge region (Figure 4a). The atom Ni2 has similar site symmetry and electronic configurations with low spin-polarization, as well as an oxidation state similar to that of the Ni1 atom. Also, its orbital contribution to the pre-edge peak is similar to that of Ni1.

For the structure S_1_, the O K-edge spectrum (calculated from the average of 63 O K-edge spectra) shows a pre-edge peak in the same energy range as in the case of S_0_, but with lower intensity. Here, nickel atoms coexist in two different geometry sites, which are a distorted trigonal bipyramid for Ni1 and a distorted tetrahedron for Ni2. The PDOS of Ni1 (spin-up) shows that the contribution to the O K pre-edge peak is mainly due to 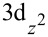, 3d*_xy_* and 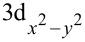 spin-down orbitals (Figure 4c). This orbital arrangement suggests that the nickel atom has an oxidation state of +3 and is in high spin-polarization with the valence electron configuration 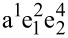 according to ligand field theory (cf. Figure 2). In the case of nickel atom Ni2, the contribution to the O K pre-edge is mainly due to 3d*_xy_*, 3d*_zx_* and 3d*_zy_* spin-down orbitals (cf. Figure 4d). In this case, the distorted tetrahedron symmetry can not explain the nickel oxidation state +4 as initially assumed. However, the electron configuration of the nickel atom is the oxidation state +3 in high spin-polarization with the valence electron configuration 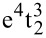 according to the ligand field theory (cf. Figure 2). This is because both e and t_2_ orbitals participate together in the transitions occurring in the pre-edge region.

When the second oxygen vacancy is introduced, that is, structure S_2_, the antiferromagnetic configuration becomes the more stable structure, and the geometry sites of the nickel atoms change to a distorted octahedron for Ni1 and a trigonal bipyramid for Ni2. The O K-edge spectrum (resulting from the average of 62 O K-edge spectra) shows a weaker pre-edge peak (in intensity) than in the case of S_0_ and S_1_. The nickel Ni1 contribution to the pre-edge peak is mainly due to 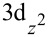 and 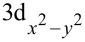 orbitals of spin-down electrons as shown in Figure 4e. Again, such an orbital configuration can not explain the oxidation state +4 for a nickel atom in octahedral coordination according to ligand field theory. However, this situation may correspond to a nickel having oxidation state +2 with the valence electron configuration 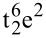 (cf. Figure 2). In the case of nickel Ni2 with spin-down electrons, the contribution to the pre-edge peak is mainly due to 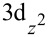 and 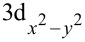 spin-up orbitals (Figure 4f), which also cannot explain the oxidation state +4 for a nickel atom in trigonal bipyramidal coordination. However, this configuration matches with a nickel atom having the oxidation state +2 in high spin-polarization, with the valence electron configuration 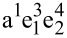 according to ligand field theory. Furthermore, the peaks in the PDOS plots (immediately above the Fermi level) that correspond to the pre-edge peaks in the O K-edge XANES spectra show sensitivity to increasing oxygen vacancy concentration. These PDOS peaks show change in intensity and splitting in response to the distortions from ideal geometric arrangements of oxygen shells surrounding the nickel atoms Ni1 and Ni2. This leads to a partial lifting of the symmetry of the ideal structures, resulting in overlap or hybridization of the d states (Figure 4).

The above analysis demonstrates that oxygen vacancies affect not only the coordination environment around nickel atoms, but also impact strongly its oxidation state. To have a deeper understanding of various contributions to the pre-edge peak with varying oxygen vacancies concentration, we focus on the O atoms neighboring nickel atoms in the three structures S_0_, S_1_, and S_2_. For these structures, we plotted the O K-edge spectrum representing the mean contributions of oxygen first nearest-neighbor shell around each of the nickel atoms Ni1 and Ni2, since the pre-edge peak originates mainly from the oxygen atoms belonging to that shell, as we demonstrated previously for Fe-doped zirconia [27]. The resulting spectra are presented in Figure 3b, where the solid and dashed lines correspond to oxygen atoms belonging the first shells around nickel atoms Ni1 and Ni2, respectively. In the case of S_0_ (black lines in Figure 3b), the contributions to the pre-edge peak correspond to two intense peaks (solid and dashed lines correspond to Ni1 and Ni2, respectively), which are mainly located at 0.82 eV above the Fermi energy. These two pre-edge peaks are almost equivalent since in S_0_, nickel atoms Ni1 and Ni2 adopt tetrahedral geometries (point group *T_d_*), with almost identical Ni–O bond lengths and small standard deviations as shown in Table 1. The latter indicates negligible distortions of the bond lengths.

**Table 1:** Ni–O bond lengths (*d_i_*, *i* = 1, 2, 3, 4, 5, 6) for nickel atoms Ni1 and Ni2 in the structures S_0_, S_1_, and S_2_ containing zero, one, and two oxygen vacancies, respectively. Ni1 and Ni2 are in tetrahedral (T), trigonal bipyramidal (TB), and octahedral (O) geometries. σ*_d_* is the standard deviation of Ni–O bond lengths for Ni1 and Ni2 within different geometries. The standard deviation increases with increasing number of oxygen vacancies, indicating how distorted the geometry becomes.

structures	S_0_		S_1_		S_2_	

oxygen vacancy	0		1		2	

nickel atom	Ni1	Ni2	Ni1	Ni2	Ni1	Ni2

geometry	T	T	TB	T	O	TB

point group	*T* *_d_*	*T* *_d_*	*D*_3_ *_h_*	*T* *_d_*	*O* *_h_*	*D*_3_ *_h_*

*d*_1_ (Å)	1.873	1.875	2.013	1.903	2.448	1.980
*d*_2_ (Å)	1.874	1.872	1.971	1.933	1.997	2.100
*d*_3_ (Å)	1.873	1.872	2.181	1.891	2.192	1.982
*d*_4_ (Å)	1.873	1.872	1.933	1.890	2.011	2.140
*d*_5_ (Å)	–	–	1.981	–	1.942	2.144
*d*_6_ (Å)	–	–	–	–	2.593	–

⟨*d*⟩ (Å)	1.873	1.873	2.016	1.904	2.197	2.069

σ*_d_*	0.00043	0.0013	0.08646	0.01737	0.24477	0.07364

In the case of structure S_1_ (red lines in Figure 3b), the contributions to the pre-edge peak are two peaks of which the first one, corresponding to Ni2, is mainly located at 0.82 eV above the Fermi energy (dashed red line), that is, at the same position than the pre-edge peaks in S_0_. This is expected since in S_1_, nickel atom Ni2 also adopts the tetrahedral geometry (point group *T_d_*). The second peak is mainly located at 1.65 eV above the Fermi energy (solid red line) and corresponds to nickel Ni1 having a trigonal bipyramidal geometry (point group *D*_3_*_h_*). However, these two peaks are less intense compared to their equivalents in S_0_. The corresponding Ni–O bond lengths have stronger variations as shown by their standard deviations presented in Table 1.

For the S_2_ structure (green lines in Figure 3b), the contributions to the pre-edge peak correspond to two peaks, of which the first one is mainly located at 0.41 eV above the Fermi energy (solid green line). The corresponding nickel atom Ni1 has an octahedral symmetry (point group *O_h_*). The second peak is mainly located at about 1.64 eV above the Fermi energy (dashed green line) and corresponds to nickel atom Ni2 in trigonal bipyramidal symmetry (point group *D*_3_*_h_*). This second peak is almost at the same position as that of Ni1 in the structure S_1_ since they have similar site symmetry. Here, nickel sites are strongly distorted since the Ni–O bond lengths for the two nickel atoms Ni1 and Ni2 present much higher variations from their equivalents in the structures S_1_ and S_0_, as shown by the corresponding standard deviations (Table 1).

The aforementioned analyses show that the combination of dopant symmetry sites and structural disorder due to the presence of oxygen vacancies in S_1_ and S_2_ may be responsible for the decrease in the pre-edge peaks with increasing concentration of oxygen vacancies.

## Conclusion

Using the density functional theory approach and X-ray absorption near-edge spectroscopy, we study the electronic structure of zirconia containing two nickel dopant atoms and oxygen vacancies at varying concentrations. The zirconia matrix contains two nickel atoms substituting two zirconium atoms at a finite separation. In our model system, the concentration of nickel dopant atoms is equivalent to 6.25 atom %. We found the two nickel atoms to be most stable when coupled together in ferromagnetic spin configurations in the absence of oxygen vacancies. Also, each of the pair of nickel atoms assumes a tetrahedral geometry in the ground state of the zirconia matrix. The oxygen K-edge XANES spectrum of this configuration shows the appearance of a pre-edge peak, which may be attributed to dipole transitions from O 1s to O 2p states that are hybridized with unoccupied Ni 3d states. However, the presence of a single oxygen vacancy in the nickel-doped zirconia matrix results in atomic relaxation and subsequent changes in the oxygen shells surrounding nickel atoms such that the site geometry of Ni1 becomes trigonal bipyramidal while that of Ni2 remains tetrahedral. The ferromagnetic configuration nevertheless remains in the ground state. Also, the oxygen K-edge XANES spectrum of this nickel-doped configuration containing a single oxygen vacancy shows a decrease in the pre-edge peak intensity. Furthermore, when two vacancies are introduced in the nickel-doped zirconia matrix, the two nickel atoms Ni1 and Ni2 have distorted octahedral and trigonal bipyramidal geometries, respectively. The ground-state configuration of this structure is antiferromagnetic. Additionally, oxygen K-edge XANES spectrum of this configuration shows a further decrease in the pre-edge peak intensity, compared to the Ni-doped configuration containing a single vacancy. Thus, the changes in the pre-edge peak evidence a major structural change in the local environment around nickel atoms and, by extension, in the zirconia matrix. Furthermore, our analyses using the XANES signatures and the projected density of states show that the oxidation state of nickel atoms change with the introduction of oxygen vacancies. The present study shows a possibility to control dopant oxidation state and magnetic order in a typical diluted magnetic oxide through the introduction of oxygen vacancies. Such a finding may be crucial for spintronics-related applications.
